# Systematic studies on the kinetic process of 20(S)-protopanaxadiol in rats and dogs: absorption, distribution, metabolism and excretion

**DOI:** 10.3389/fphar.2024.1430780

**Published:** 2024-06-20

**Authors:** Pengfei Li, Min Zhang, Meng Chen, Guangxu Liu, Linghui Meng, Dan Zhang

**Affiliations:** ^1^ Department of Pharmacy, Beijing Anding Hospital, National Clinical Research Center for Mental Disorders, National Center for Mental Disorders, Beijing Key Laboratory of Mental Disorders, Capital Medical University, Beijing, China; ^2^ Advanced Innovation Center for Human Brain Protection, Capital Medical University, Beijing, China; ^3^ Department of Research Ward, Beijing Chao-Yang Hospital, Capital Medical University, Beijing, China; ^4^ Department of Clinical Pharmacology, Aerospace Center Hospital, Beijing, China

**Keywords:** 20(S)-protopanaxadiol, pharmacokinetics, metabolize, ginseng, unity of medicine and food

## Abstract

**Background and Objective:**

Ginseng has been regarded as a precious medicinal herb with miraculous effects in Eastern culture. The primary chemical constituents of ginseng are saponins, and the physiological activities of ginsenosides determine their edible and medicinal value. The aim of this study is to comprehensively and systematically investigate the kinetic processes of 20(S)—protopanaxadiol (PPD) in rats and dogs, in order to promote the rational combination of ginseng as a drug and dietary ingredient.

**Methods:**

PPD was administered, and drug concentration in different biological samples were detected by liquid chromatography tandem mass spectrometry (LC/MS/MS) and radioactive tracer methods. Pharmacokinetic parameters such as absorption, bioavailability, tissue distribution, plasma protein binding rate, excretion rate, and cumulative excretion were calculated, along with inference of major metabolites.

**Results:**

This study systematically investigated the absorption, distribution, metabolism, excretion (ADME) of PPD in rats and dogs for the first time. The bioavailabilities of PPD were relatively low, with oral absorption nearly complete, and the majority underwent first-pass metabolism. PPD had a high plasma protein binding rate and was relatively evenly distributed in the body. Following oral administration, PPD underwent extensive metabolism, potentially involving one structural transformation and three hydroxylation reactions. The metabolites were primarily excreted through feces and urine, indicating the presence of enterohepatic circulation. The pharmacokinetic processes of PPD following intravenous administration aligned well with a three-compartment model. In contrast, after gastric administration, it fitted better with a two-compartment model, conforming to linear pharmacokinetics and proportional elimination. There were evident interspecies differences between rats and dogs regarding PPD, but individual variations of this drug were minimal within the same species.

**Conclusion:**

This study systematically studied the kinetic process of PPD in rats and also investigated the kinetic characteristics of PPD in dogs for the first time. These findings lay the foundation for further research on the dietary nutrition and pharmacological effects of PPD.

## Introduction

Since ancient times, ginseng has been regarded as a precious medicinal herb with miraculous effects in Eastern culture. Its unique appearance and profound therapeutic properties have made it a symbol of health in people’s minds. In traditional Chinese medicine, ginseng is believed to possess multiple benefits, such as replenishing qi, nourishing blood, calming the mind, and enhancing cognitive function (Hasegawa, 2004). It is used for treating various diseases, including cardiovascular disorders, diabetes, and cancer (Cho et al., 2001; Lee et al., 2005; Huang et al., 2022). However, besides its medicinal value, ginseng also holds significant culinary value (Luo et al., 2021; Lv et al., 2023). In daily cuisine, ginseng can be used for simmering soups, brewing teas, and making pastries. This culinary application allows individuals to enjoy delicious food and ginseng’s nutritional components, embodying the concept of “the unity of medicine and food.” The cure of these diseases and nutritional components are closely associated with the chemical composition of ginseng.

**FIGURE 1:**
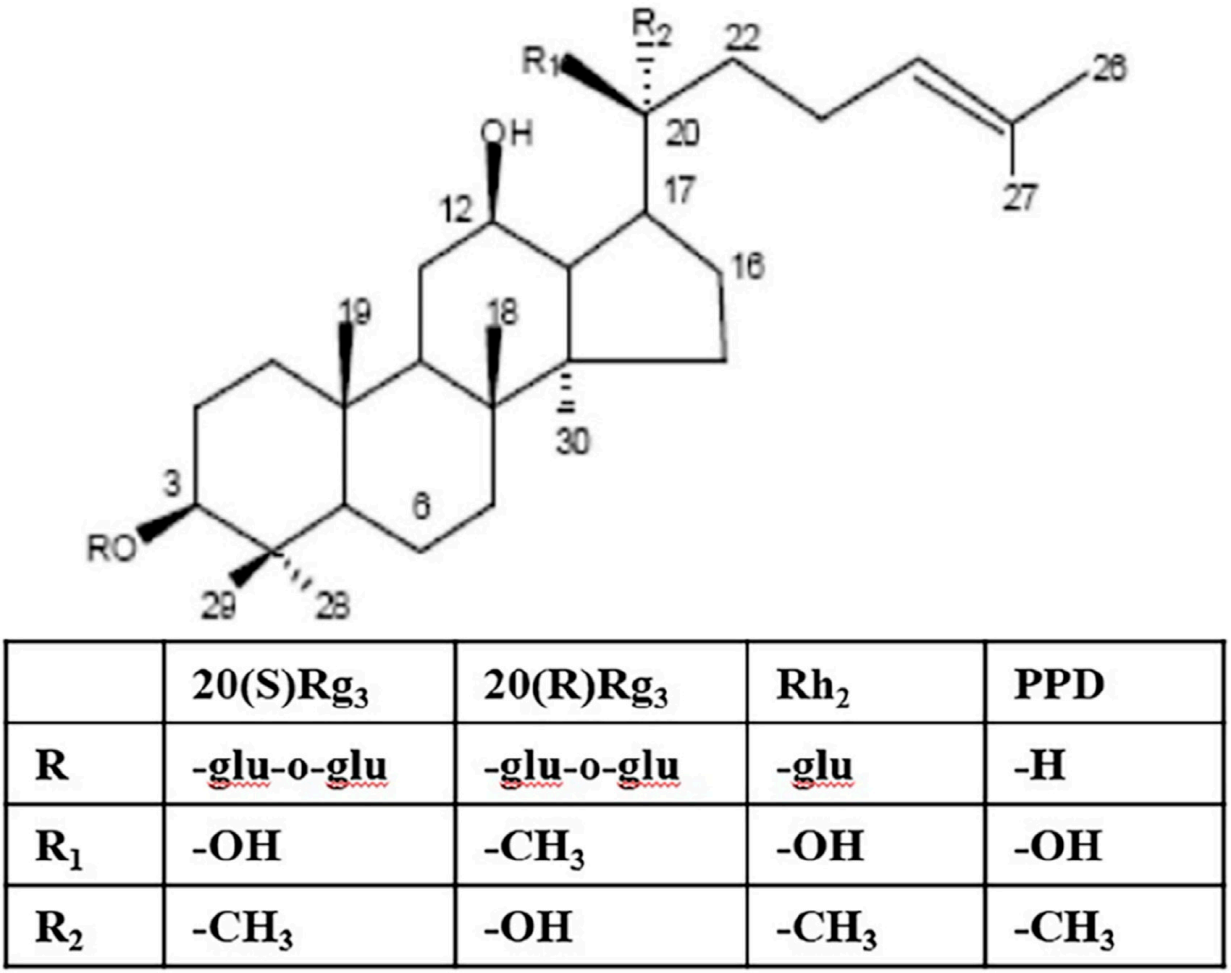
Structures of PPD and ginsenoside Rh_2_ (internal standard).

The primary chemical constituents of ginseng are saponins, and the physiological activities of ginsenosides determine their edible and medicinal value (Hu et al., 2022). Dammarane saponins and their metabolites are the primary sources of its activity. Dammarane saponins isolated from Panax ginseng can be classified into two categories based on their aglycone moieties: protopanaxadiol saponins (e.g., ginsenosides Ra_1_, Rb_1_, Rb_2_, Rb_3_, Rg_3_, Rh_2_, Rc, Rd) and protopanaxatriol saponins (e.g., ginsenosides Re, Rg_1_, Rg_2_, Rh_1_) (Jin et al., 2019). Protopanaxadiol saponins Rg_3_ and Rh_2_ inhibit tumor cell proliferation and growth and induce differentiation and apoptosis *in vitro* and *in vivo*. 20(S)-protopanaxadiol (PPD) is the aglycone of protopanaxadiol saponins and exhibits more potent activity than Rg_3_ and Rh_2_ (Figure 1) (Tanaka et al., 1966).

There are numerous reports on the utilization of the LC/MS/MS method to detect ginsenosides (Choi et al., 2016; Jin et al., 2019; Yang et al., 2019), panaxadiol (Cai et al., 2013), and PPD, including quantitative detection in rat plasma (Ren et al., 2008; Han et al., 2010; Wang et al., 2012; Bao et al., 2013; Zhao et al., 2016; Jin-Qi et al., 2018) as well as in human plasma and urine (Zhang et al., 2009; Choi et al., 2016). The above articles, which focus on establishing quantitative methods, often mention the use of narrow pharmacokinetic studies (only in plasma, not involving tissue distribution, excretion, and metabolism). For instance, Ling-Ti Kong et al. conducted pharmacokinetic studies on two structurally similar saponins, protopanaxatriol and PPD, in rats (Kong et al., 2013). Lei Xu et al. reported gender differences in tissue distribution, excretion, metabolism, and anti-inflammatory studies of panaxadiol in rats, focusing on gender differences and anti-inflammatory effects (Xu et al., 2022). Currently, no comprehensive and systematic study has been conducted on the absorption, distribution, metabolism, and excretion patterns of PPD *in vivo*, and no studies on the *in vivo* process of PPD in dogs have been found.

This study aims to explore the process and quantitative changes of PPD in rats and dogs, elucidating its absorption, distribution, biotransformation, and excretion processes to facilitate the rational combination of ginseng as a medicinal and dietary ingredient.

## Methods

### Experimental animals

Wistar rats (220–270 g) were provided by the Animal Laboratory of Shenyang Pharmaceutical University. Beagle dogs (8–12 kg) were sourced from the Experimental Animal Center of the Academy of Military Medical Sciences. All animal experiments followed the instructions of the Chinese Physiological Society Regulations for the Management of Laboratory Animals and were approved by the Ethics Committee for the Care and Use of Laboratory Animals of Jilin University.

### Study design, drug administration and biological sample collection and processing

The grouping, administration, and sample collection for PPD are summarized in Table 1. In rats, 0.5 mL of venous blood was collected from the retro-orbital plexus after administration, while in dogs, 1.0 mL of blood was obtained from the central vein of the forelimb. The blood samples were collected in heparinized tubes, and centrifuged (3,000 rpm) for 10 min to separate the plasma. Tissue and fecal samples were weighed, and methanol was added at a rate of 3 mL·g⁻^1^, homogenized, sonicated, and centrifuged to obtain the supernatant. Urine and bile samples were stored directly. All samples were stored at −20°C until analysis.

**TABLE 1:** Grouping, administration, and sample collection of PPD in rats and dogs.

	Groups	Number	Dose	Administration	Sample collection
1	Rat venous PK	6	17.5 mg·kg^-1^	Caudal vein injection	Collect venous blood before and after administration at 0.25, 0.5, 1.0, 2.0, 4.0, 6.0, 8.0, 10.0, 12.0, 24.0 h
2	Rat gavage PK	18	17.5, 35.0, 70.0 mg·kg^-1^	Gavage	Collect venous blood at 0.5, 1.0, 2.0, 3.0, 4.0, 6.0, 8.0, 10.0, 12.0, 24.0 h before and after administration
3	Canine venous PK	6	5 mg·kg^-1^	Injection into the lateral saphenous vein of the hind limbs	Collect venous blood before and after administration at 0.17, 0.25, 0.5, 1.0, 1.5, 2.0, 3.0, 4.0, 6.0, 8.0, 12.0, and 24.0 h
4	Canine gavage PK	18	5, 10, 20 mg·kg^-1^	Gavage	Collect venous blood before and after administration at 0.25, 0.5, 1.0, 1.5, 2.0, 3.0, 4.0, 6.0, 8.0, 10.0, 12.0, and 24.0 h
5	Rat tissue distribution	32	35 mg·kg^-1^	Gavage	Immediately after administration, dissect and collect tissues such as brain, heart, lungs, liver, spleen, kidney, gastric wall, fat, testes, ovaries, bladder, pancreas, uterus, small intestine wall, skeletal muscle, abdominal wall smooth muscle, gastric and intestinal contents at various time points
6	Rat bile excretion	8	35 mg·kg^-1^	Gavage	After the implementation of bile intubation surgery, collect bile samples from various time periods of 0–1, 1–3, 3–6, 6–9, 9–12, 12–24, 24–36 h before and after administration, and record the volume
7	Rat urine and fecal excretion	8	35 mg·kg^-1^	Gavage	Place in a metabolic cage and collect urine and fecal samples at different time periods of 0–4, 4–8, 8–12, 12–24, 24–36, 36–48, and 48–72 h before and after administration. Record the volume of urine samples and weigh the fecal samples
8	Rat bile excretion by radiotracer	6	5.55 MBq·kg^-1^	Gavage	Collect bile samples from different time periods of 0–1, 1–3, 3–6, 6–9, 9–12, 12–24, 24–36 h before and after administration, and record the volume
9	Rat urine and fecal excretion by radiotracer	6	5.55 MBq·kg^-1^	Gavage	Collect urine samples and fecal samples from 0 to 4, 4 to 8, 8 to 12, 12 to 24, 24 to 48, 48–72 h, 72–96 h, 96–144 h after administration. Urine sample recording volume, fecal sample weighing

Note: Randomly average grouping was used between each experimental group, with half male and half female in each dose group; The intravenous group ate and drank freely, while the gavage group fasted for 12 h, drank freely, and ate 2 h after administration; The rat tissue distribution group was divided into 2, 4, 8, and 24 h groups according to time; The tracer method group received a dosage of 5.55 MBq · kg^-1^, of [^3^H] PPD.

### LC/MS/MS quantitative analysis method

A methanol-water mixture (1:1, v/v) of 100 μL was added to biological sample (50 μL of rat plasma, dog plasma, fecal supernatant, or 100 μL of tissue supernatant, bile or urine). Additionally, 100 μL of internal standard solution (500 ng·mL^−1^ Rh2) and 50 μL of sodium hydroxide solution (0.3 mol·L^−1^) were mixed into the sample. The mixture was vortexed for 1 min and oscillated for 15 min. After centrifugation at 3,000 rpm for 5 min, the supernatant was separated and evaporated under an air stream at 40°C. The residue was dissolved in 300 μL of mobile phase, and 20 μL of the solution was injected for LC/MS/MS analysis.

Chromatographic analysis was conducted using an Agilent 1100 high-performance liquid chromatography system (Agilent Technologies, United States) and a Zorbax Extend C_18_ column (50 mm × 2.1 mm, 3.5 μm). The mobile phase consisted of a mixture of methanol, acetonitrile, and a 10 mmol/L solution of acetic acid (45:45:10, v/v/v) at a flow rate of 0.4 mL·min^−1^. The column temperature was set at 40°C.

The mass spectrometry analysis was performed using an API 4000 triple quadrupole mass spectrometer equipped with an electrospray ionization source (ESI) and Analyst 1.3 data system (Applied Biosystems, United States). The ion spray voltage was set at 4800 V, and the temperature was maintained at 320°C. The nitrogen flow rate was as follows: sheath gas at 276 kPa, nebulize gas at 173 kPa, and curtain gas at 69 kPa, collision gas at 28 kPa. This method used positive ionization and multiple reaction monitoring (MRM) with DP voltage at 35 V. The specific ion reactions for quantification were *m/z* 461.6 → *m/z* 425.5 (PPD) and *m/z* 623.50 → *m/z* 605.5 (internal standard, Rh_2_).

### Quantitative analysis of PPD excretion using the radiotracer method

Urine and bile were directly measured for radioactivity after dilution 1:1 with ethanol. After natural drying, the feces were weighed and ground into powder. A 1:12.5 fecal homogenate was prepared with anhydrous ethanol, and the supernatant was centrifuged to determine its radioactivity. Measurement was performed using the homogeneous method with a 1217 RACKBETA liquid scintillation counter (LKB, Sweden) (Klinger et al., 1996).

### Plasma protein binding assay

The plasma protein binding rate of PPD was determined using the equilibrium dialysis technique. A certain concentration of fresh rat plasma test solution was prepared, and 1.2 mL of each sample was placed into a tubular semi-permeable dialysis bag measuring 10 cm in length. The bag was tightly sealed at both ends and placed in a stoppered test tube containing 4 mL of dialysis solution. The drug concentrations used in this experiment were 200, 500, and 1000 ng·mL^−1^. Three parallel samples were prepared for each concentration. After adding the test drug, the test tubes were placed in a 37°C water bath and continuously agitated for 24 h. The concentrations of PPD in both the plasma inside the bag and the dialysate outside the bag were measured.

### PPD metabolism study

Considering that PPD and its metabolites typically contain several hydroxyl groups, and their secondary mass spectra commonly exhibit water loss, the present study utilized MRM analysis to analyze potential metabolites of PPD. Cumulative bile samples were collected from rats before and 0–12 h after oral administration. Following solid-phase extraction, LC/MS/MS was employed to investigate the metabolic products.

### Data processing

The plasma drug concentrations in rats and dogs were measured using LC/MS/MS, and the concentration-time curves were plotted. Topfit 2.0 pharmacokinetic software (Thomae GmbH, Germany) was utilized to calculate pharmacokinetic parameters using a non-compartmental model. The main parameters included peak concentration (C_max_), time to reach peak concentration (T_max_), area under the concentration-time curve (AUC), elimination half-life (t_1/2_), clearance rate (CL), apparent volume of distribution (V_d_), and others. A bar chart was generated to illustrate the distribution of drug concentrations in different tissues at various time points after administration.

The excretion rate, cumulative excretion, and cumulative excretion percentage of PPD in urine, feces, and bile were calculated. After administration, the cumulative excretion curves of PPD in urine, feces, and bile were plotted. The radioactivity and cumulative excretion percentage of [^3^H] PPD were calculated using the same method.

The [^3^H] radioactivity in rat urine, feces, and bile was calculated based on the working curve of the day. The cumulative excretion of the radioactive dose and the cumulative excretion percentage in urine, feces, and bile were determined. After administration, the cumulative excretion curves of [^3^H] radioactivity in urine, feces, and bile were plotted.

## Results

### Blood concentration and pharmacokinetic parameters

**FIGURE 2:**
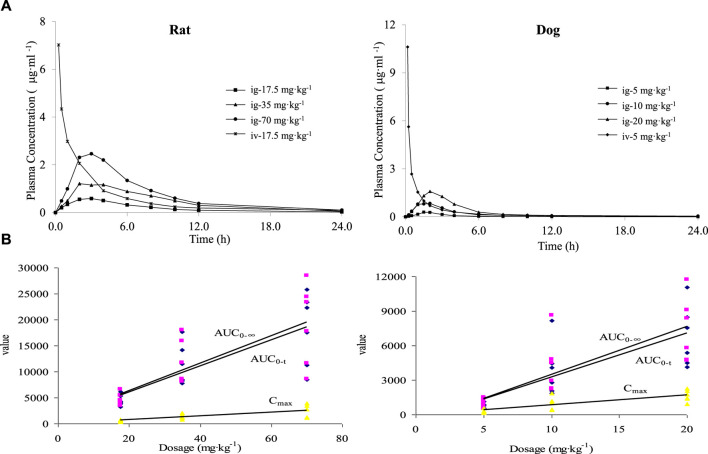
Mean blood concentration time curve **(A)** and dose linear relationship **(B)** of PPD in rats and dogs.

After intravenous administration of PPD to rats at a dose of 17.5 mg·kg^−1^ and oral gavage administration at doses of 17.5, 35, and 70 mg·kg^−1^, the average blood concentration-time curve is shown in Figure 2A. The main pharmacokinetic parameters are presented in Table 2. The dynamic process of PPD in rats after intravenous administration fitted well with a three-compartment model, while the dynamic process after oral gavage administration fitted well with a two-compartment model. After oral gavage administration of PPD to rats, pharmacokinetic parameters such as AUC_0-t_, AUC_0-∞_, and C_max_ showed a linear correlation with the dose (r > 0.708, *p* < 0.001). The relationship curves are shown in Figure 2B. The average absolute bioavailability of rats was 28.5%.

**TABLE 2:** Main pharmacokinetic parameters after intravenous and gavage administration in rats and dogs.

Rat					
Dose groups	mg·kg^-1^	17.5 iv	17.5 ig	35 ig	70 ig
C_max_	μg·mL^-1^	—	0.608 ± 0.164	1.36 ± 0.48	2.57 ± 1.13
T_max_	h	—	2.83 ± 0.75	3.00 ± 0.89	3.33 ± 0.52
t_1/2_	h	8.99 ± 1.64	6.07 ± 1.47	4.38 ± 1.61	5.06 ± 1.31
AUC_0-t_	μg·h·mL^-1^	15.80 ± 6.78	4.50 ± 1.08	11.31 ± 3.97	18.15 ± 6.97
AUC_0-∞_	μg·h·mL^-1^	17.09 ± 7.49	4.78 ± 1.12	11.78 ± 4.31	19.06 ± 7.78
CL	mL·min^-1^·kg^-1^	20.48 ± 10.00	63.58 ± 13.74	54.85 ± 17.77	73.32 ± 37.30
V_d_	L·kg^-1^	15.60 ± 7.06	33.67 ± 11.31	19.82 ± 6.63	29.45 ± 9.50

Dog					
Dose groups	mg·kg^-1^	5 iv	5 ig	10 ig	20 ig
C_max_	μg·mL^-1^	—	0.299 ± 0.158	1.02 ± 0.53	1.66 ± 0.48
T_max_	h	—	1.67 ± 0.41	1.58 ± 0.49	2.50 ± 0.55
t_1/2_	h	6.25 ± 1.81	4.23 ± 1.58	5.66 ± 1.55	8.56 ± 1.35
AUC_0-t_	μg·h·mL^-1^	8.06 ± 2.42	0.887 ± 0.366	3.89 ± 2.33	6.85 ± 2.68
AUC_0-∞_	μg·h·mL^-1^	8.32 ± 2.58	0.904 ± 0.372	4.23 ± 2.42	7.41 ± 2.81
CL	mL·min^-1^·kg^-1^	13.05 ± 4.05	126.6 ± 48.7	59.35 ± 27.35	60.68 ± 21.77
V_d_	L·kg^-1^	6.74 ± 1.63	41.12 ± 7.60	29.90 ± 17.30	45.63 ± 20.51

The blood concentration-time curve after intravenous administration of PPD to dogs at a dose of 5 mg·kg^−1^ and oral gavage administration at doses of 5, 10, and 20 mg·kg^−1^ is depicted in Figure 2A. The major pharmacokinetic parameters are listed in Table 2. The kinetics of PPD in dogs after intravenous administration fitted well with a three-compartment model, while after oral gavage administration, it fitted well with a two-compartment model, consistent with the findings in rats. Following oral gavage administration of PPD to dogs, pharmacokinetic parameters including AUC_0-t_, AUC_0-∞_, and C_max_ also exhibited a linear correlation with the dose (r > 0.708, *p* < 0.001). The corresponding relationship curves are shown in Figure 2B. The average absolute bioavailability of PPD in dogs was 11.0%.

### Plasma protein binding rate

At three concentration levels of 200, 500, and 1,000 ng/mL, the plasma protein binding rate of PPD at 24 h was 97.56% ± 1.48%, 97.95% ± 1.90%, and 98.56% ± 0.82%, respectively. PPD exhibited a high plasma protein binding rate, averaging 98.02%, indicating that PPD was likely to predominantly exist in a protein-bound state in the blood after drug absorption.

### Tissue distribution of PPD

**FIGURE 3:**
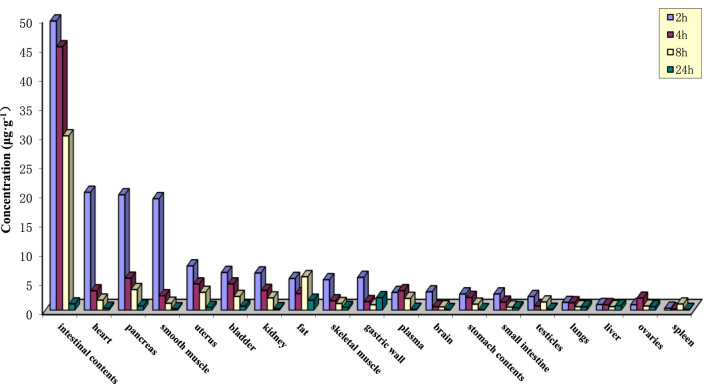
Tissue drug concentrations at various time points after intragastric administration of PPD in rats.

After oral gavage administration of 35 mg·kg^−1^ PPD to rats, the distribution of the drug in 18 different tissues and plasma at various time points is depicted in Figure 3. The top five tissues in terms of concentration at 2, 4, 8, and 24 h were as follows: After 24 h post-administration, except for spleen, liver, lung, small intestine, gastric wall, ovary, and bladder, the levels of PPD in other tissues were less than 10% of the levels observed at 2 h, which suggested that PPD was not prone to accumulate significantly in various tissues.

### The excretion of PPD

**FIGURE 4:**
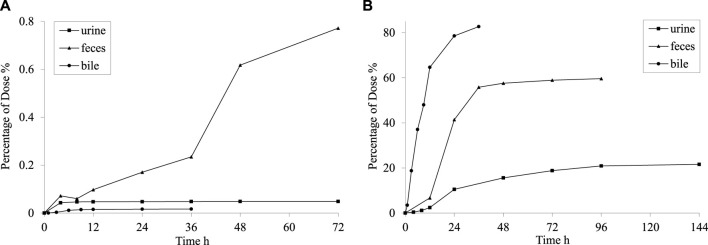
Mean cumulative excretion curve after intragastric administration of PPD **(A)** and [^3^H] PPD **(B)** in rats.

Following the oral administration of 35 mg·kg^−1^ PPD to rats, the percentage of PPD relative to the administered dose was 0.02% ± 0.01% in bile at 36 h, 0.05% ± 0.07% in urine at 72 h, and 0.77% ± 0.41% in feces at 72 h. The average cumulative excretion curve is displayed in Figure 4A. The levels of unchanged PPD in urine, feces, and bile were relatively low in rats, suggesting extensive drug metabolism within the rat body.

After orally administering 5.55 MBq/kg [^3^H] PPD to rats, the percentage of cumulative excretion of [^3^H] in relation to the administered dose was found to be 82.7% ± 7.9% in bile at 36 h, 59.6% ± 7.1% in feces at 96 h, and 21.6% ± 2.9% in urine at 144 h. The average cumulative excretion curve is shown in Figure 4B. These findings indicated that the drug’s metabolites could be excreted through feces and urine, with fecal excretion being the main route of elimination. The comparison between the percentages in bile at 36 h and feces at 96 h indicated the presence of hepatic enterohepatic circulation, where some metabolites might undergo intestinal reabsorption before being slowly eliminated through urine.

### PPD metabolism

**FIGURE 5:**
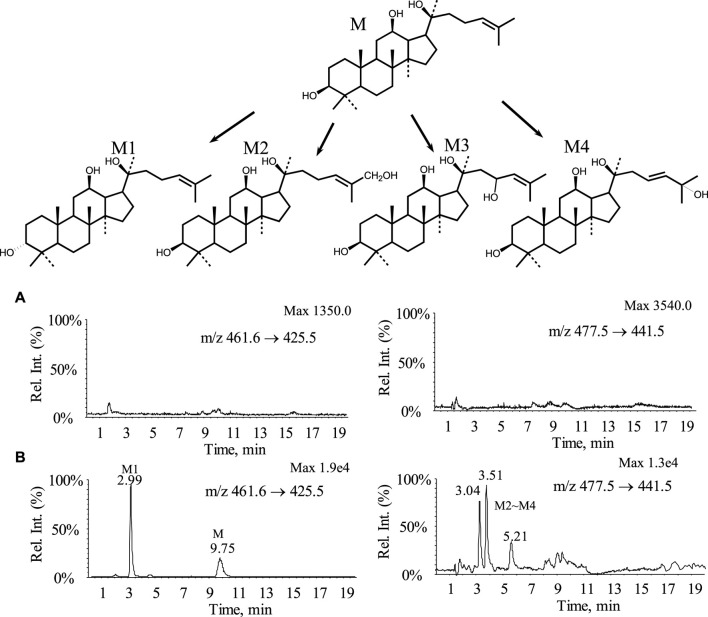
MRM chromatograms and possible metabolic pathways of bile samples from rats before and after administration **(A)** Blank bile sample; **(B)** Bile samples from rats 0–12 h after administration.

By comparing the MRM chromatograms of rat bile samples obtained before and after administration, it was observed that several new chromatographic peaks appeared in the post-dose samples besides the detectable parent drug peak. The corresponding chromatograms can be seen in Figure 5, indicating the presence of distinct metabolites. Parent drug M: Retention time of 9.7 min, which was consistent with the peak of the standard. Metabolite M1: A new peak was observed at a retention time of 3.0 min. The molecular weight and mass spectra fragmentation ions for this peak were identical to those of the parent drug, suggesting that it might be an isomeric metabolite resulting from double bond isomerization or a configuration change in one carbon atom of three chiral centers. Metabolites M2∼M4 ([M + H]^+^ = 477): By selectively monitoring *m/z* 477.5 → 441.5, three peaks appeared at retention times of 3.04, 3.51, and 5.21 min. These peaks corresponded to compounds with a molecular weight 16 Da higher than the parent drug, indicating hydroxylation metabolites.

Metabolic experiments revealed that the parent drug underwent extensive metabolism, including one conformational conversion and three hydroxylation metabolites. All four metabolites’ polarity was higher than the parent drug, while no phase II conjugated metabolites were detected. Possible metabolic pathways are shown in Figure 5. The results from the metabolic experiments aligned with the excretion study findings.

## Discussion

The AUC reflected the extent of absorption. In this study, the absolute bioavailability of PPD in rats and dogs was 28.5% and 11.0%, respectively, which indicated that the bioavailability of PPD was relatively low but did not necessarily imply poor oral absorption. Combining the results of [^3^H] radiotracer excretion for PPD suggested that oral absorption of PPD was relatively complete, with at least 82.7% entering the body. It could be inferred that most of it (approximately 82.7% minus 28.5%) underwent first-pass metabolism and existed in the body as metabolites. T_max_ and Ka reflected the rate of absorption. After oral administration to rats and dogs, T_max_ for absorption was relatively slow in both species, with dogs exhibiting a significantly faster absorption rate than rats. According to the “Lipinski’s rule of 5” (Lipinski et al., 2001; Lipinski et al., 1997), the approaching complete absorption of PPD was attributed to its low molecular weight. In contrast, the slow absorption was primarily influenced by its low water solubility and hydrogen bonding capability.

The V_d_ values for rats and dogs were found to be 15.60 ± 7.06 and 6.74 ± 1.63 L·kg^−1^, respectively, which indicated that PPD was evenly distributed in the bodies of both rats and dogs, with relatively equal distribution in blood and various tissues. There was no significant local accumulation phenomenon, which closely corresponds with the subsequent tissue distribution results. The tissue distribution results indicated relatively high levels of PPD in intestinal contents, heart, pancreas, and abdominal smooth muscle within 2 h of administration. However, PPD was rapidly eliminated as time progressed, and minimal accumulation was observed in the various tissues. The plasma protein binding rate of PPD at three concentration levels remained above 97% for 24 h, indicating that PPD primarily existed in a protein-bound state in the blood after absorption.

Drug elimination typically involved excretion and metabolic processes. t_1/2_ and CL reflected the elimination rate of the parent drug. In rats and dogs, the t_1/2_ and CL values indicated the parent drug’s relatively slow excretion rate, primarily attributed to its low water solubility and limited glomerular permeability. Excretion studies using [^3^H]-labelled PPD revealed extensive metabolism of PPD following oral administration. The metabolites were primarily excreted via feces and urine. Some metabolites underwent intestinal reabsorption, followed by slow elimination through urine, indicating the presence of enterohepatic circulation. Preliminary metabolic experiments showed widespread metabolism of the parent drug, including one configurational conversion and three hydroxylation metabolites. All four metabolites exhibited increased polarity compared to the parent drug, while no phase II conjugated metabolites were detected.

In the case of PPD administration in rats and dogs, significant differences were observed in AUC, V_d_, and t_1/2_. However, within each species, the CV for AUC, V_d_, and t_1/2_ was less than 50%, indicating minor individual variation but notable inter-species differences.

Pharmacokinetic models were mathematical simulations established to quantitatively study the rate characteristics of drug processes within the body. Commonly used models included compartmental models and elimination kinetics models. When fitting the blood concentration of PPD after intravenous and oral administration in rats and dogs, a three-compartment model exhibited good agreement with the kinetic processes of PPD in the body after intravenous administration. In contrast, a two-compartment model showed a better fit for the kinetic processes of PPD after oral administration. This suggests that compared to oral administration, the absorption, distribution, and elimination of PPD were more complex after intravenous administration. After administering PPD orally to rats and dogs at low, medium, and high doses, the pharmacokinetic parameters AUC_0-t_, AUC_0-∞_, and C_max_ showed a linear correlation with the dose administered, which indicated that PPD followed linear pharmacokinetics within the three dosage ranges, characterized by first-order elimination kinetics or constant-rate elimination.

## Conclusion

This study comprehensively and systematically studied the kinetic process of PPD in rats and also investigated the kinetic characteristics of PPD in dogs for the first time. PPD exhibited a relatively low bioavailability, approaching complete oral absorption but substantial first-pass metabolism. It displayed a high plasma protein binding rate and relatively uniform distribution throughout the body. The metabolites were mainly excreted via feces and urine, indicating the presence of enterohepatic circulation. The kinetic processes of PPD following intravenous administration fitted well with a three-compartment model, and after gavage administration conformed well to a two-compartment model, consistent with a linear pharmacokinetic constant ratio elimination model. There were significant interspecies differences between rats and dogs regarding PPD, whereas individual differences within the same species were minor. These findings lay the foundation for further research on the dietary nutrition and pharmacological effects of PPD.

## Data Availability

The original contributions presented in the study are included in the article/Supplementary Material, further inquiries can be directed to the corresponding authors.
